# Real-world treatment of over 1600 Japanese patients with *EGFR* mutation-positive non-small cell lung cancer with daily afatinib

**DOI:** 10.1007/s10147-019-01439-5

**Published:** 2019-04-05

**Authors:** Kazuo Tamura, Toshihiro Nukiwa, Akihiko Gemma, Nobuyuki Yamamoto, Masaya Mizushima, Kaori Ochai, Rie Ikeda, Hisaya Azuma, Yoichi Nakanishi

**Affiliations:** 1 General Medical Research Center, School of Medicine, Fukuoka University, Fukuoka, Japan; 2 Tohoku University, Miyagi, Japan; 3 Nippon Medical School, Tokyo, Japan; 4 Division of Pulmonology and Medical Oncology, Third Department of Internal Medicine, Wakayama Medical University, Wakayama, Japan; 5 Speciality Care Medicine, Medical Division, Nippon Boehringer Ingelheim Co., Ltd., Tokyo, Japan; 6 PMS Division, Statistics Analysis Department 2, EPS Corporation, Tokyo, Japan; 7 Post Marketing Surveillance Group, Pharmacovigilance, Nippon Boehringer Ingelheim Co., Ltd., Tokyo, Japan; 8 Research Institute for Diseases of the Chest, Graduate School of Medical Sciences, Kyusyu University, Fukuoka, Japan

**Keywords:** Afatinib, *EGFR*m + NSCLC, Japanese patients, Real-world data, Post-marketing observational study

## Abstract

**Background:**

This prospective, post-marketing observational study in Japanese patients aimed to evaluate the safety and effectiveness of daily afatinib use in general practice.

**Methods:**

This non-interventional study (NCT02131259) enrolled treatment-naïve and pre-treated patients with inoperable/recurrent *EGFR* mutation-positive NSCLC, eligible for afatinib treatment as per the afatinib label in Japan. Patients received afatinib at the approved dose (20, 30, 40, or 50 mg/day; physician decision), and were observed following treatment initiation for 52 weeks or until premature discontinuation. Primary endpoint was the incidence of adverse drug reactions (ADRs). Secondary endpoints included ADRs of special interest, and objective response rate (ORR). Post hoc Cox multivariate analyses were used to assess prognostic factors associated with the incidence of ADRs.

**Results:**

1602 patients, at 374 sites (April 2014–March 2015), were included in the analysis; 307 (19%) were aged ≥ 75 years. The most frequently reported ADRs (all/grade 3–4) were diarrhea (78%/15%), rash/acne (59%/6%), stomatitis (31%/4%), and nail effects (38%/4%). Serious ADRs resulting in death occurred in 18 patients (1%). 762 patients (48%) had ≥ 1 afatinib dose reduction and 366 (23%) discontinued due to ADRs; the most common reason for both was diarrhea (8.2% and 6.7%, respectively). ORR was 40.1%.

**Conclusions:**

Real-world treatment of 1602 Japanese patients with afatinib was associated with a predictable ADR profile. Afatinib showed effectiveness in inoperable/recurrent *EGFR* mutation-positive NSCLC, especially as first-line treatment. As with other EGFR TKIs, prompt management of adverse events is needed in the Japanese population, to reduce serious events and outcomes, including interstitial lung disease.

**Electronic supplementary material:**

The online version of this article (10.1007/s10147-019-01439-5) contains supplementary material, which is available to authorized users.

## Introduction

Mutations of the epidermal growth factor receptor *(EGFR)* gene are important drivers of non-small cell lung cancer (NSCLC). The frequency of *EGFR* mutations is higher in Asian than Caucasian populations; in Japanese NSCLC patients, the prevalence of *EGFR* mutations has been reported to be approximately 30–40% [1].

Standard first-line treatment for advanced NSCLC harboring an *EGFR* mutation is an EGFR tyrosine kinase inhibitor (TKI). Currently, approved first-line agents include the first-generation EGFR TKIs erlotinib and gefitinib, which reversibly inhibit EGFR, and the second-generation ErbB family blocker afatinib, which irreversibly blocks signaling from all members of the ErbB family: EGFR (ErbB1), HER2 (ErbB2), ErbB3, and ErbB4 [2]. Afatinib was previously assessed in two pivotal phase 3 trials: LUX-Lung 3 (conducted globally) and LUX-Lung 6 (conducted in China, Thailand and South Korea), which showed that afatinib significantly improved progression-free survival (PFS) versus chemotherapy in patients with treatment-naïve *EGFR* mutation-positive NSCLC [3, 4]. Also, in both trials, overall survival was found to be improved by afatinib in patients with *EGFR* Del19 mutation-positive NSCLC (pre-specified analyses) [5].

The LUX-Lung 3 trial enrolled 83 patients from Japan; outcomes for the Japanese subgroup patients were generally consistent with those for the overall population [6]. In a pre-planned subgroup analysis of Japanese patients in LUX-Lung 3 (of whom 54 received afatinib and 29 cisplatin/pemetrexed chemotherapy), PFS was found to be significantly longer with afatinib than with cisplatin/pemetrexed [median 13.8 versus 6.9 months; hazard ratio (HR), 0.38, 95% confidence interval (CI) 0.20–0.70; *p* = 0.0014], with more pronounced improvements among patients with common mutations (Del19/L858R: HR, 0.28, 95% CI 0.15–0.52; *p* < 0.0001) and specifically Del19 mutations (HR, 0.16, 95% CI 0.06–0.39; *p* < 0.0001). Furthermore, in patients harboring a Del19 mutation, median overall survival was also significantly longer with afatinib than with cisplatin/pemetrexed (46.9 versus 31.5 months; HR, 0.34, 95% CI 0.13–0.87; *p* = 0.0181) [6].

Based on these results, in 2014, afatinib was approved in Japan for the treatment of patients with inoperable or recurrent *EGFR* mutation-positive NSCLC [7, 8]. However, at the time of approval, only limited data were available on the use of afatinib in Japanese clinical practice settings. The prevalence of *EGFR* mutations in Japanese patients has been found to be greater than in other populations, and the safety profile of afatinib may differ slightly from that in other populations [1]. The incidence of interstitial lung disease (ILD) is also higher in Japan than in other countries, and in LUX-Lung 3, a higher frequency of adverse events was reported in the Japanese subgroup than in the overall trial population [3, 6, 9–12]. We, therefore, initiated this prospective, post-marketing observational study to evaluate the safety and effectiveness of daily afatinib used in day-to-day clinical practice in Japan. Of note, as there are no restrictions to the label in Japan, afatinib can be used across multiple treatment lines and for both EGFR TKI-naïve and EGFR TKI-pre-treated patients. Consequently, in contrast to the LUX-Lung 3 trial, which included only treatment-naïve patients, this study reflects real treatment outcomes achieved with afatinib during daily clinical practice in Japan. Additionally, in accordance with the Japanese Good Post-Marketing Study Practice (GPSP) regulations, all patients treated with afatinib were enrolled in the study, including both EGFR TKI-naïve and EGFR TKI-pre-treated patients, eliminating the selection bias seen in randomized clinical trials.

## Patients and methods

### Study design and patients

This non-interventional, observational study aimed to investigate the safety and effectiveness of 1 year of afatinib treatment in Japanese NSCLC patients. Patients with inoperable or recurrent *EGFR* mutation-positive NSCLC were eligible for afatinib treatment (in accordance with the afatinib label in Japan); no specific enrollment criteria were applied in terms of demographics or baseline characteristics. Patients received afatinib at the approved dose (20, 30, or 40 mg/day), based on physician decision; dose reduction was permitted in the case of adverse events (AEs), and dose escalation up to 50 mg/day was permitted following at least 3 weeks of treatment at 40 mg/day with acceptable toxicity.

Patients were selected for inclusion using the continuous investigation system, a method of registration in which all patients who start treatment are enrolled in the study continuously (without exception) until the planned total number of patients is reached. Patients were observed following treatment initiation for 52 weeks or until premature discontinuation.

This post-marketing study was conducted in accordance with the Japanese GPSP regulations and Japanese Good Vigilance Practice (GVP) regulations; the trial was registered at clinical trials.gov: NCT02131259. The study was carried out in routine clinical practice and no interventions were made for the purpose of the study; therefore, in accordance with GPSP regulations, written informed consent of the patients was not required.

### Endpoints and assessments

Observations were made before the first dose of afatinib (baseline) and after 4, 12, 26, 40, and 52 weeks, or on discontinuation of afatinib.

The primary outcome measure was the incidence of adverse drug reactions (ADRs). An ADR was defined as an AE for which the investigator or the sponsor (or both) assessed the causal relationship to afatinib as ‘yes’, ‘probably yes’ or ‘can’t be denied’. For each AE, the investigators recorded the time of onset and end, intensity, seriousness, outcome, causal relationship, and action taken with afatinib; the intensity of each AE was determined according to the Common Terminology Criteria for Adverse Events (CTCAE) version 3.0.

The safety outcome measure used as a secondary endpoint was the number of ADRs of special interest (specifically, diarrhea, rash/acne, nail effects, and ILD). In the event of the occurrence of any of these ADRs at grade 3 or higher, the investigators collected additional information including details of the clinical course of the event in question and the results of imaging tests and laboratory tests.

The effectiveness outcome measure used as a secondary endpoint was the objective response rate (ORR) based on physicians’ assessment. Objective response was defined as a complete or partial response, evaluated with reference to the Response Evaluation Criteria in Solid Tumors (RECIST) version 1.1. Information was collected from the attending physicians for all patients, irrespective of evaluation criteria. Tumor progression was also evaluated by the physician, using radiologic assessment and/or clinical judgement.

An independent committee, the ‘Afatinib Appropriate Use Review Committee’, reviewed all assessments conducted for the purpose of the study every 6 months until the study ended.

### Statistical analysis

To achieve 95% probability of detecting an ADR with a true incidence of 0.20% or more in at least one patient (or 99% probability of detecting an ADR with a true incidence of 0.30% or more), it was necessary to enroll at least 1500 patients. This incidence rate (0.30%) was chosen as it is equal to the incidence of grade ≥ 3 ILD reported in clinical studies. Reported incidence rates of all other ADRs of special interest (diarrhea, rash/acne, and nail effects) are greater than 0.30%.

Data were included for all patients who received afatinib during the follow-up period. Safety was evaluated using the ‘safety set’, which included all patients who received treatment and had at least one observation. It was not possible to identify an ‘effectiveness set’ of patients with tumor assessments according to RECIST; therefore, all patients in the safety set were included to avoid overestimation of effectiveness.

Post hoc Cox multivariate analyses were used to assess the relationship between potential prognostic factors and the incidence of ADRs. To select the factors for inclusion in each multivariate analysis, univariate logistic regression analysis was initially performed to assess the relationship between each baseline factor and the incidence of any grade or grade ≥ 3 ADRs, and ADRs of special interest (diarrhea, rash/acne, nail effects and ILD). Cox multivariate regression analyses were then performed for the baseline factors that had an odds ratio and 95% confidence interval less than one, or greater than one, by the stepwise method. In case of factors with multi-collinearity, one representative factor was selected. Cox multivariate regression analyses were not performed for grade ≥ 3 ILD due to the low incidence of these ADRs.

## Results

### Patients

A total of 1602 patients were enrolled at 374 sites between April 2014 and March 2015 and were included in the analysis. Baseline demographic and clinical characteristics are shown in Table 1. Almost all (99%) patients had an *EGFR* mutation. A majority of patients had adenocarcinoma (97%), and 86% of patients had an Eastern Cooperative Oncology Group performance status (ECOG PS) of 0 or 1. More than half (55%) of patients had two or more lines of previous chemotherapy, and 59% of patients had received prior EGFR TKI treatment.

**Table 1:** Baseline demographic and clinical characteristics (*N *= 1602)

Characteristic	
Gender, *n* (%)	
Male	655 (40.9)
Female	947 (59.1)
Median age, years (range)	67 (34–90)
Age class 1, years, *n* (%)	
< 65	629 (39.3)
≥ 65	970 (60.6)
Missing	3 (0.2)
Age class 2, years, *n* (%)	
< 75	1292 (80.7)
≥ 75	307 (19.2)
Missing	3 (0.2)
BMI class, kg/m^2^, *n* (%)	
< 25	1324 (82.7)
≥ 25	254 (15.9)
Missing	24 (1.5)
BSA class, m^2^, *n* (%)	
< 1.52	733 (45.8)
≥ 1.52	845 (52.8)
Missing	24 (1.5)
Smoking history, *n* (%)	
Never smoked	935 (58.4)
Ex-smoker	630 (39.3)
Current smoker	25 (1.6)
Unknown	12 (0.8)
ECOG PS, *n* (%)	
0	642 (40.1)
1	739 (46.1)
2	143 (8.9)
3	63 (3.9)
4	15 (0.9)
*EGFR* mutation status, *n* (%)	
Any	1578 (98.5)
Del19	1020 (63.7)
L858R	421 (26.3)
T790M	65 (4.1)
Tumor histology, *n* (%)	
Adenocarcinoma	1554 (97.0)
Squamous cell carcinoma	14 (0.9)
Large cell carcinoma	1 (0.1)
Mixed	9 (0.6)
Other	23 (1.4)
Missing	1 (0.1)
Clinical stage, *n* (%)	
IIIB	94 (5.9)
IV	1206 (75.3)
Other	301 (18.8)
Missing	1 (0.1)
Presence of bone metastasis, *n* (%)	
Yes	638 (39.8)
No	964 (60.2)
Presence of contralateral lung metastasis, *n* (%)	
Yes	452 (28.2)
No	1150 (71.8)
Previous diagnosis of cardiac disorder, *n* (%)	
Yes	23 (1.4)
No	1576 (98.4)
Unknown	3 (0.2)
Concomitant cardiac disorder, *n* (%)	
Yes	84 (5.2)
No	1515 (94.6)
Unknown	3 (0.2)
Previous diagnosis of gastrointestinal disorder, *n* (%)	
Yes	73 (4.6)
No	1526 (95.3)
Unknown	3 (0.2)
Concomitant gastrointestinal disorder, *n* (%)	
Yes	134 (8.4)
No	1465 (91.5)
Unknown	3 (0.2)
Afatinib starting dose, mg, *n* (%)	
20	115 (7.2)
30	246 (15.4)
40	1241 (77.5)
Total dose taken, mg, *n* (%)	
< 280	31 (1.9)
280 to < 1120	280 (17.5)
1120 to < 6720	735 (45.9)
≥ 6720	553 (34.5)
Unknown	3 (0.2)
Number of previous chemotherapies, *n* (%)	
0	486 (30.3)
1	241 (15.0)
2	274 (17.1)
≥3	601 (37.5)
Previous radiotherapy within 1 year, *n* (%)	
Yes	108 (6.74)
No	1494 (93.3)
Prior EGFR TKI, *n* (%)	
Yes	948 (59.2)
No	654 (40.8)

*BMI* body mass index, *BSA*, body surface area, *ECOG PS* Eastern Cooperative Oncology Group performance score, *EGFR* epidermal growth factor receptor, *TKI* tyrosine kinase inhibitor

The majority of patients received a starting dose of 40 mg/day. Low-dose treatment initiation was observed at a higher rate in females, versus males, and in patients with lower versus higher body weights (Table 2). There was no significant difference in dose selection between patients with good versus poor baseline performance status.

**Table 2:** Dose of first, and last, intake by gender, baseline body weight, and baseline ECOG PS

	Dose of first intake				Dose of last intake^a^			
	Patients (*N*)	20 mg, *n* (%)	30 mg, *n* (%)	40 mg, *n* (%)	20 mg, *n* (%)	30 mg, *n* (%)	40 mg, *n* (%)	50 mg, *n* (%)
All	1602	115 (7.2)	246 (15.4)	1241 (77.5)	454 (28.3)	554 (34.6)	580 (36.2)	13 (0.8)
Gender								
Male	655	28 (4.3)	85 (13.0)	542 (82.7)	129 (19.7)	222 (33.9)	298 (45.5)	6 (0.9)
Female	947	87 (9.2)	161 (17.0)	699 (73.8)	325 (34.3)	332 (35.1)	282 (29.8)	7 (0.7)
Body weight (kg)								
<50	606	68 (11.2)	123 (20.3)	415 (68.5)	198 (32.7)	214 (35.3)	189 (31.2)	4 (0.7)
50 to < 60	513	33 (6.4)	76 (14.8)	404 (78.8)	160 (31.2)	184 (35.9)	167 (32.6)	2 (0.4)
60 to < 70	307	10 (3.3)	38 (12.4)	259 (84.4)	64 (20.8)	110 (35.8)	130 (42.3)	3 (1.0)
≥70	169	4 (2.4)	9 (5.3)	156 (92.3)	30 (17.8)	44 (26.0)	91 (53.8)	4 (2.4)
ECOG PS								
0	642	33 (5.1)	78 (12.1)	531 (82.7)	191 (29.8)	218 (34.0)	226 (35.2)	7 (1.1)
1	739	54 (7.3)	110 (14.9)	575 (77.8)	208 (28.1)	257 (34.8)	267 (36.1)	6 (0.8)
2	143	18 (12.6)	39 (27.3)	86 (60.1)	36 (25.2)	50 (35.0)	57 (39.9)	0
3	63	8 (12.7)	17 (27.0)	38 (60.3)	14 (22.2)	25 (39.7)	24 (38.1)	0
4	15	2 (13.3)	2 (13.3)	11 (73.3)	5 (33.3)	4 (26.7)	6 (40.0)	0

*ECOG PS,* Eastern Cooperative Oncology Group performance status

^a^One patient, a female with body weight < 50 kg and ECOG PS of 1, received a last dose of afatinib of 10 mg

### Safety

#### Incidence of ADRs and serious ADRs

ADRs of any grade, and of grade 3 or higher, that occurred in ≥ 5% of patients are shown in Table 3. The most frequently reported ADRs (all grades/grade 3–4) were diarrhea (78%/15%), and rash/acne (59%/6%), nail effects (38%/4%), and stomatitis (32%/4%) (grouped terms). Serious ADRs occurred in 337 patients (21%); the most frequently reported serious ADRs were diarrhea (*n* = 117, 7%) and ILD (*n* = 60, 4%). Serious ADRs resulting in death occurred in 18 patients (1%).

**Table 3:** All-grade and grade ≥ 3^a^ rates of the most commonly reported^b^ ADRs (excluding malignant neoplasm progression)

	Safety set (*n* = 1602)	
	All	Grade ≥ 3
Any ADR, *n* (%)	1509 (94.2)	577 (36.0)
Diarrhea	1257 (78.5)	242 (15.1)
Rash/acne^c^	938 (58.6)	93 (5.8)
Nail effects^c^	602 (37.6)	65 (4.1)
Stomatitis^c^	512 (32.0)	62 (3.9)
Decreased appetite	220 (13.7)	76 (4.7)
Nausea	122 (7.6)	16 (1.0)
Vomiting	90 (5.6)	16 (1.0)
Dry skin	83 (5.2)	3 (0.2)

*ADR* adverse drug reactions

^a^Graded according to Common Terminology Criteria for Adverse Events (NCI-CTCAE) version 3.0

^b^Events were included if reported in ≥ 5% of patients overall

^c^Grouped term

#### ADRs of special interest

Incidences and times to onset of ADRs of special interest are shown in Table 4; median time to onset for diarrhea, rash/acne, and stomatitis was less than 2 weeks.

**Table 4:** Incidence and time to onset for ADRs of special interest

	Patients, *n* (%)	Median time to onset, days (range)
Diarrhea	1257 (78.5)	5 (1–316)
Rash/acne^a^	938 (58.6)	11 (1–406)
Nail effects^a^	602 (37.6)	38 (1–526)
Stomatitis^a^	512 (32.0)	9 (1–327)
ILD^a^	70 (4.4)	35.5 (3–329)

*ADR* adverse drug reactions, *ILD* interstitial lung disease

^a^Grouped term

ILD, including ILD-like events, occurred in 70 patients (4%) [grade 3–4: 28 (2%) patients; grade 5: 12 (1%) patients]; ‘real’ ILD occurred in 60 patients overall (4%). Six patients (< 1%) had an increase in plasma creatinine concentration following grade ≥ 3 diarrhea.

**Fig. 1:**
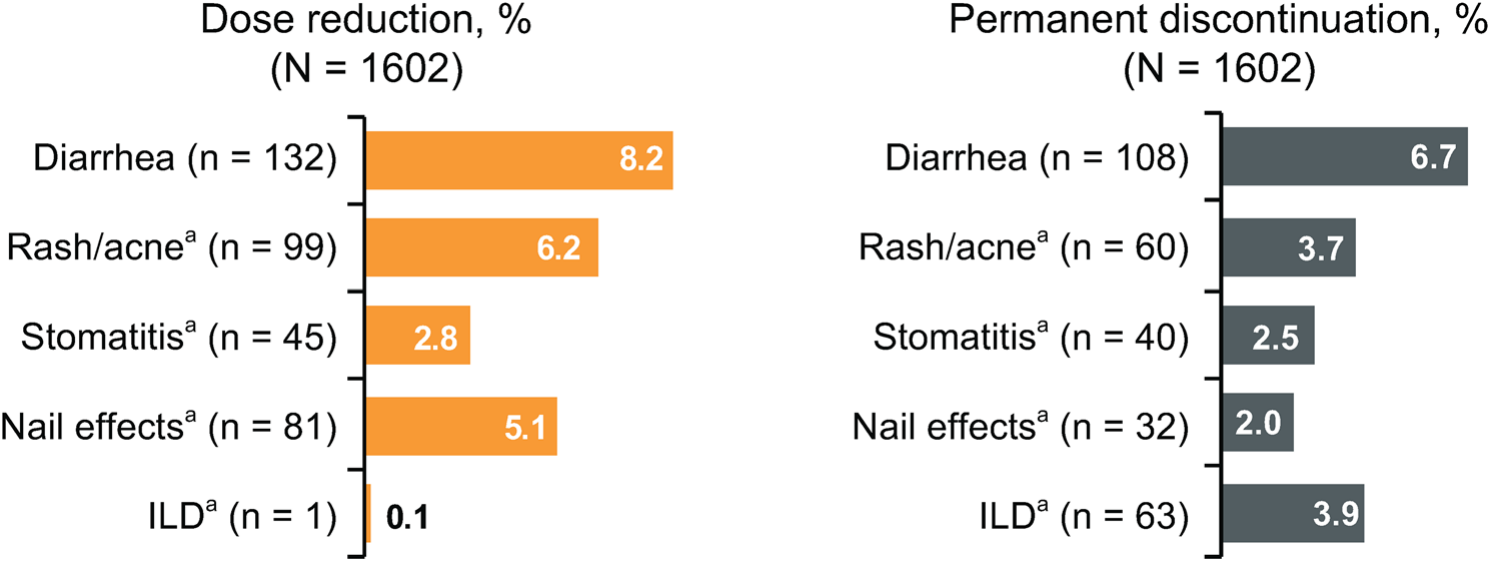
Dose reductions and discontinuations due to ADRs of special interest. *ADR* adverse drug reaction, *ILD* interstitial lung disease. ^a^Grouped term

Almost a half of the patients (*n* = 762, 48%) had ≥ 1 afatinib dose reduction and 366 patients (23%) discontinued afatinib due to ADRs. Dose reductions and discontinuations due to ADRs of special interest are shown in Fig. 1. The most common reason for dose reduction and for permanent discontinuation of afatinib was diarrhea (in 8.2% and 6.7% of patients, respectively).

#### Factors associated with ADRs

**Fig. 2:**
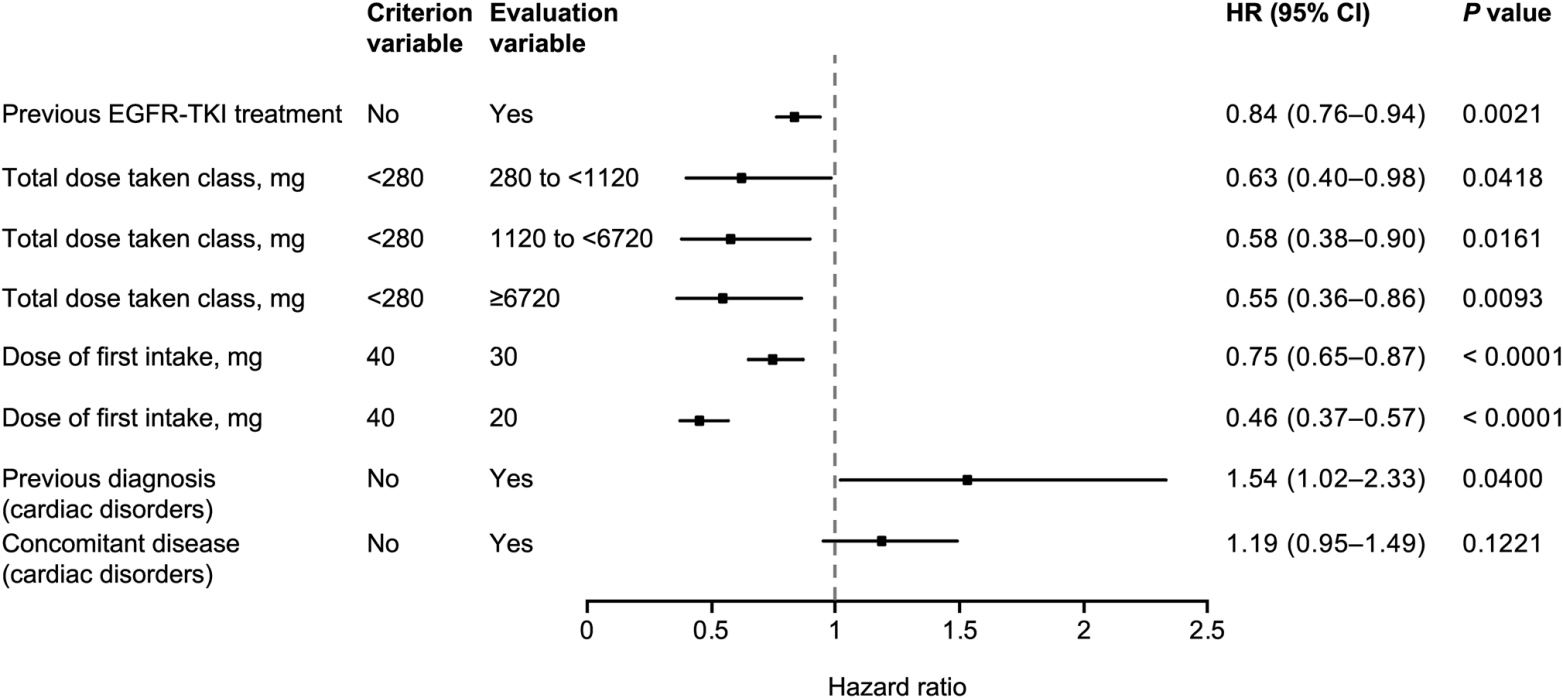
Cox multivariate analysis of factors affecting the incidence of ADRs (excluding progressive disease) (*N* = 1595). *ADR* adverse drug reaction, *CI* confidence interval, *EGFR* epidermal growth factor receptor, *HR* hazard ratio, *TKI* tyrosine kinase inhibitor

**Fig. 3:**
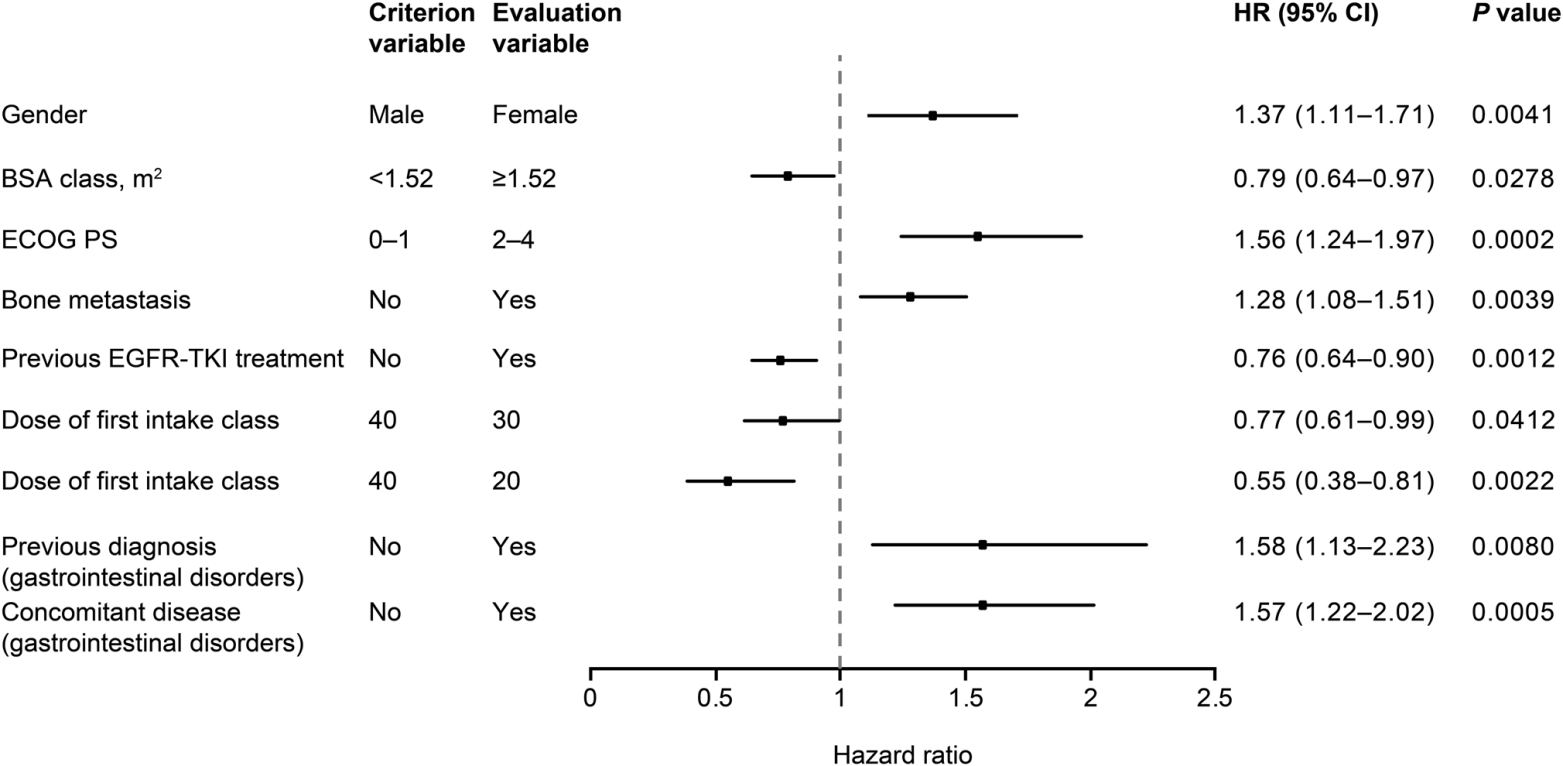
Cox multivariate analysis of factors affecting the incidence of grade ≥ 3 ADRs (excluding progressive disease) (*N* = 1575). *BSA* body surface area, *CI* confidence interval, *ECOG PS* Eastern Cooperative Oncology Group performance score, *EGFR* epidermal growth factor receptor, *HR* hazard ratio, *TKI* tyrosine kinase inhibitor

The results of Cox multivariate analyses of the impact of different factors on the incidence of ADRs and of grade ≥ 3 ADRs are shown in Figs. 2 and 3, respectively. The starting dose of afatinib was found to have a significant impact on the incidence of ADRs, with lower starting dose favored by the HRs; for patients who received a starting dose of 20 mg compared to 40 mg, the HR was 0.46 (95% CI 0.37–0.57; *p* < 0.0001) for all ADRs and 0.55 (95% CI 0.38–0.81; *p* = 0.0022) for grade ≥ 3 ADRs. Previous EGFR TKI treatment was associated with a lower risk of ADRs, whereas ECOG PS 2–4, female gender, bone metastasis, and previous and concomitant gastrointestinal disorders were all associated with a significantly higher risk of grade ≥ 3 ADRs.

**Fig. 4:**
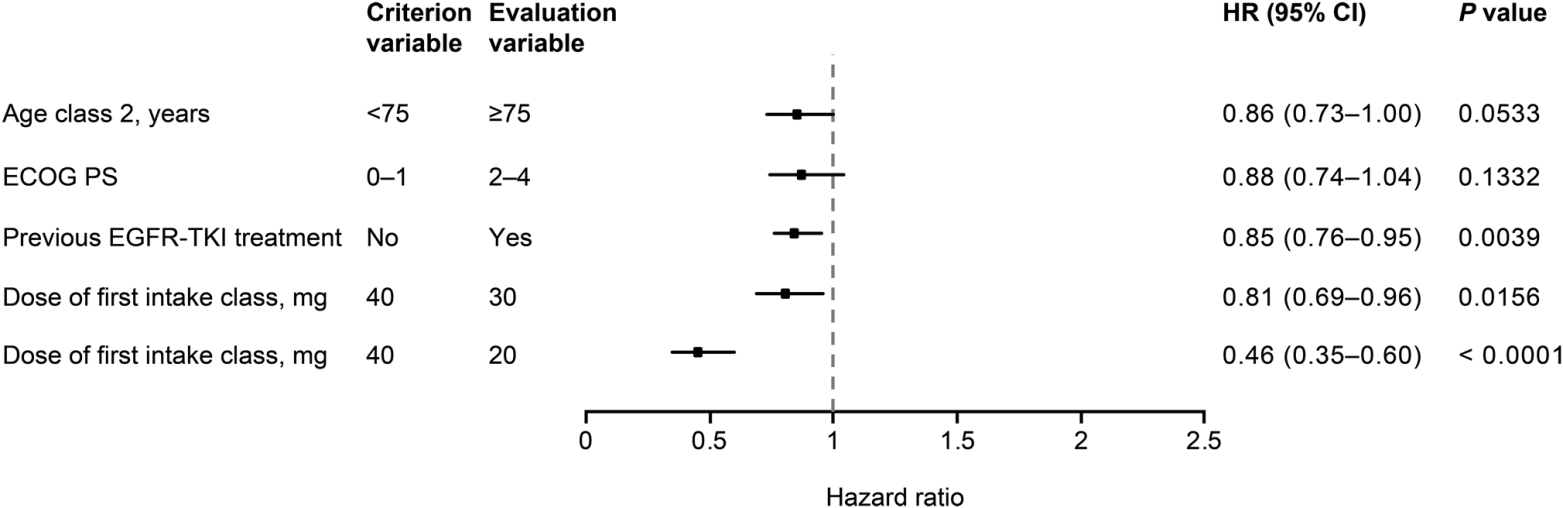
Cox multivariate analysis of factors affecting the incidence of diarrhea (*N* = 1596). *CI* confidence interval, *ECOG PS* Eastern Cooperative Oncology Group performance score, *EGFR* epidermal growth factor receptor, *HR* hazard ratio, *TKI* tyrosine kinase inhibitor

**Fig. 5:**
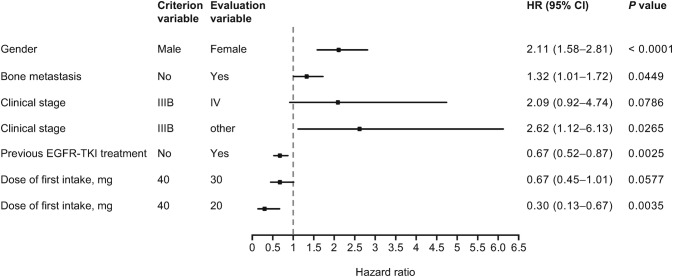
Cox multivariate analysis of factors affecting the incidence of grade ≥ 3 diarrhea (*N* = 1574). *CI* confidence interval, *EGFR* epidermal growth factor receptor, *HR* hazard ratio, *TKI* tyrosine kinase inhibitor

The results of Cox multivariate analyses of the impact of factors on the incidence of all grades and of grade ≥ 3 diarrhea are shown in Figs. 4 and 5, respectively. Incidence of diarrhea was not significantly affected by age (< 75 years versus ≥ 75 years), or ECOG PS (0–1 versus 2–4); however, starting dose, gender, and previous EGFR TKI treatment were found to have a significant impact on the incidence of grade ≥ 3 diarrhea.

**Fig. 6:**
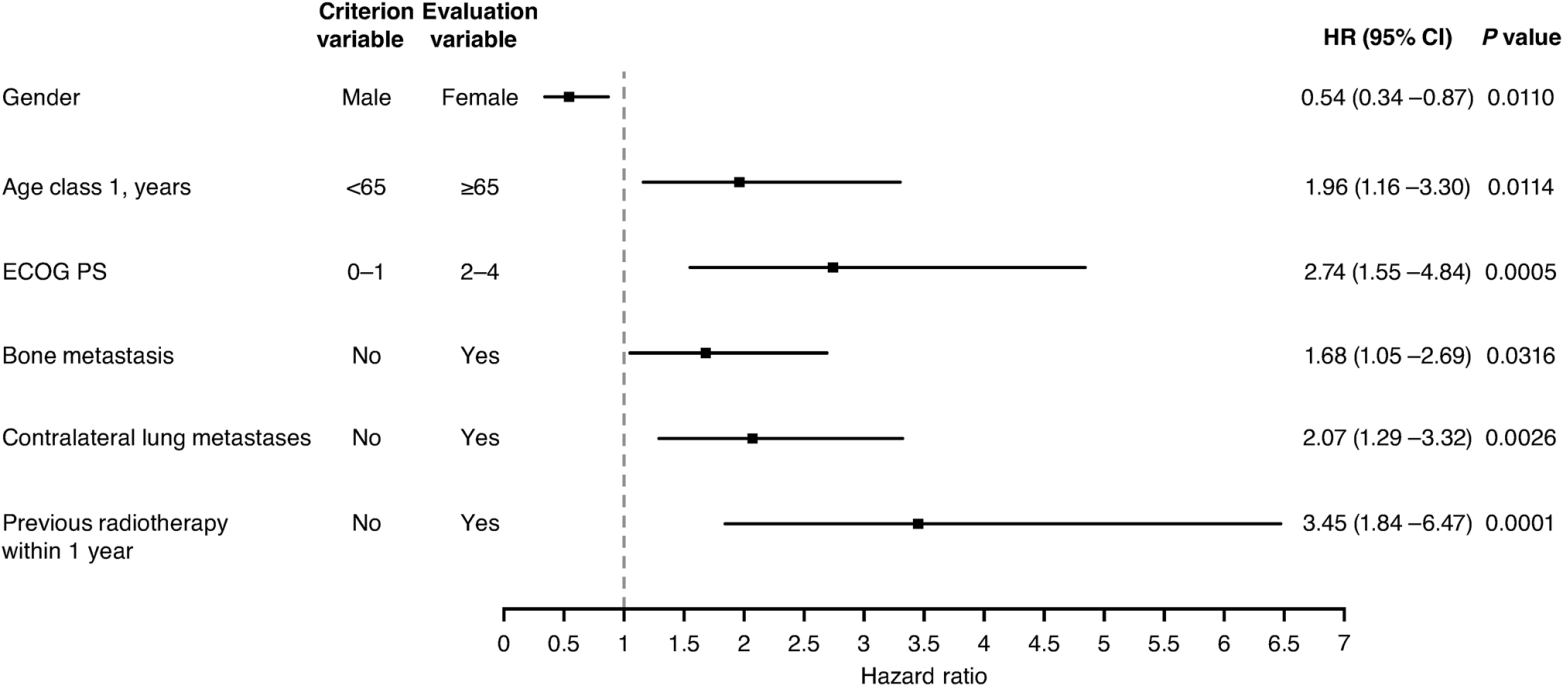
Cox multivariate analysis of factors affecting the incidence of ILD (*N* = 1587). *CI* confidence interval, *ECOG PS* Eastern Cooperative Oncology Group performance score, *HR* hazard ratio, *ILD* interstitial lung disease

The results of Cox multivariate analyses of the impact of factors on the incidence of ILD are shown in Fig. 6. Females had a lower risk of ILD compared to males (HR, 0.54, 95% CI 0.34–0.87; *p* = 0.0110). A significantly greater risk of ILD was associated with ECOG PS 2–4 compared to 0–1 (HR, 2.74, 95% CI 1.55–4.84; *p* = 0.0005), presence of contralateral lung metastases (HR, 2.07, 95% CI 1.29–3.32; *p* = 0.0026), and previous radiotherapy within 1 year (HR, 3.45, 95% CI 1.84–6.47; *p* = 0.0001).

In addition to Cox multivariate analyses, comparison of ADRs (including malignant neoplasm) by age group identified no differences for any grade/grade ≥ 3 ADRs, including ADRs of special interest, between patients aged < 75 years and those aged ≥ 75 years (Supplementary Table).

### Effectiveness

The ORR was 40.1% (642 of 1602 patients), and is shown for different subgroups in Table 5. Compared with the EGFR TKI-pre-treated subgroup, a greater proportion of the EGFR TKI-naïve subgroup achieved an objective response. For patients who received a starting dose of 40 mg, the ORR was 68.4% in EGFR TKI-naïve patients, compared with 21.3% in those who had previously received an EGFR TKI. It is of note that use of a lower starting dose did not negatively affect response rates (in either EGFR TKI-pre-treated or EGFR TKI-naïve patients).

**Table 5:** ORR by subgroup

	All patients	*N*	Patients with objective response, *n* (%)
		1602	642 (40.1)
EGFR TKI-pre-treated patients			
Starting dose, mg	20	81	18 (22.2)
	30	176	34 (19.3)
	40	691	147 (21.3)
EGFR TKI-naïve patients			
Starting dose, mg	20	34	24 (70.6)
	30	70	43 (61.4)
	40	550	376 (68.4)
Previously untreated patients			
< 75 years			
Starting dose, mg	20	9	7 (77.8)
	30	26	18 (69.2)
	40	362	269 (74.3)
≥ 75 years			
Starting dose, mg	20	18	12 (66.7)
	30	21	16 (76.2)
	40	49	35 (71.4)

*EGFR* epidermal growth factor receptor, *ORR* objective response rate, *TKI* tyrosine kinase inhibitor

For previously untreated patients, age (< 75 years or ≥ 75 years) did not affect ORR. For patients aged ≥ 75 years who received afatinib as first-line treatment with a starting dose of 30 mg, ORR was 76.2%.

## Discussion

The results of this post-marketing, observational study provide long-term safety and effectiveness data for 1602 Japanese patients with *EGFR* mutation-positive NSCLC treated with afatinib in routine clinical practice.

No specific patient-selection criteria were applied to enroll patients in the study, and all patients treated with afatinib (as decided by their physician) were included in the analysis. Consequently, this study population is likely to be highly representative of the NSCLC patient population in Japan. It is of note that the median age of patients in this study was 67 years, which is 5 years older than that of the LUX-Lung 3 study population [3]. The method of enrollment should also avoid the bias inherent in clinical study populations, which typically exclude patients with a poor ECOG PS, a history of previous EGFR TKI treatment, or significant comorbidities. These routine exclusion criteria often also limit the number of older patients included in clinical trials; only 4% of the LUX-Lung 3 study population were ≥ 75 years of age, compared to 19% of the current study population [13].

Lower initial starting doses were observed at a higher rate in female patients and in patients with lower body weight. Reasons for the choice of initial starting dose were not collected in this study; however, it can be assumed that physicians may have started patients on a reduced dose of afatinib to avoid toxicity due to potentially increased exposure to the drug [14]. Dose reductions occurred at a higher frequency in females and in patients with lower body weights, which is similar to the results seen in the LUX-Lung 3 and 6 studies, and in the global real-world observational study, RealGiDo [15, 16].

The types and frequencies of ADRs reported in Japanese patients were consistent with the known safety profile of afatinib. The most frequently reported ADRs were the same as those reported for the Japanese population in the LUX-Lung 3 trial, namely diarrhea, rash/acne, nail effects, and stomatitis, and the frequencies of ADRs of special interest were consistent with previous findings in clinical trials with afatinib [3, 4, 6]. Median time to onset of ADRs such as diarrhea, rash/acne, and stomatitis was less than 2 weeks, highlighting the importance of monitoring patients during the early course of treatment. It is also important for physicians to be aware of any potential preventative measures, such as educating patients on skin and oral care, and use of dietary adaptations including the avoidance of heavy stimulating meals. Additionally, as demonstrated by the LUX-Lung 3 and 6 studies, these ADRs can be effectively managed with supportive care (including anti-diarrheal medication) and dose adjustments [15]. In LUX-Lung 3 and 6, tolerability-guided dose adjustment was found to reduce the frequency/intensity of AEs without affecting the efficacy of afatinib [15]. In the case of severe diarrhea, it is especially important to intervene early by initiating intravenous hydration to prevent dehydration and subsequent renal failure, which may otherwise prove fatal.

ILD has been reported to occur in Japanese patients with all currently available EGFR TKIs, at a higher incidence than reported outside of Japan [3, 9–12]. In the current study, the incidence of ILD during afatinib treatment was consistent with previous findings. The risk of ILD was higher in males, which again is consistent with previous findings [17]. ILD can be fatal; therefore, it is important to carefully monitor patients receiving EGFR TKIs (particularly Japanese patients) and to treat ILD early to prevent progression, including cessation of the drug.

The ORR in EGFR TKI-naïve patients (67.7%) was consistent with that reported in randomized clinical trials of afatinib (61–74%; investigator assessed) [3, 4, 6]. Of course, this observational study has a number of inherent limitations; notably, tumor response was evaluated by the primary care physician (and not by external reviewers), and the RECIST criteria were not used for the assessment of response in all patients. Nevertheless, this post-marketing surveillance study provided an opportunity to assess response outcomes with afatinib in the real-world setting, which differs in a number of aspects from the clinical trial setting. Consistent with previously reported data, response rates were lower in patients who had previously received EGFR TKIs [18]. Response rates were not notably affected by the starting dose, supporting the physician’s selection of the appropriate dose of afatinib, following meticulous assessment of each patient. As previously noted in clinical trials with afatinib, advanced age (≥ 75 years) did not adversely affect the clinical benefits to patients in this study, suggesting afatinib can be an effective and tolerable treatment for elderly patients in the Japanese population with *EGFR* mutation-positive NSCLC [13].

In conclusion, this large, post-marketing observational study, involving more than 1600 Japanese patients in a real-world clinical setting, showed the ADR profile of afatinib to be predictable and consistent with that reported in clinical trial settings, with the majority of patients able to continue treatment. Afatinib showed effectiveness in inoperable/recurrent *EGFR* mutation-positive NSCLC, especially as a first-line treatment; outcomes were comparable to those seen in randomized clinical trials. As observed for other EGFR TKIs, during treatment with afatinib, AEs such as diarrhea and ILD need to be managed early in Japanese patients, to reduce serious events and outcomes.

## Electronic supplementary material

Below is the link to the electronic supplementary material.

Supplementary material 1 (DOCX 12 kb)
