# Correction to: Real-world treatment of over 1600 Japanese patients with EGFR mutation-positive non-small cell lung cancer with daily afatinib

**DOI:** 10.1007/s10147-019-01488-w

**Published:** 2019-06-25

**Authors:** Kazuo Tamura, Toshihiro Nukiwa, Akihiko Gemma, Nobuyuki Yamamoto, Masaya Mizushima, Kaori Ochai, Rie Ikeda, Hisaya Azuma, Yoichi Nakanishi

**Affiliations:** 1 General Medical Research Center, School of Medicine, Fukuoka University, Fukuoka, Japan; 2 Tohoku University, Miyagi, Japan; 3 Nippon Medical School, Tokyo, Japan; 4 Division of Pulmonology and Medical Oncology, Third Department of Internal Medicine, Wakayama Medical University, Wakayama, Japan; 5 Speciality Care Medicine, Medical Division, Nippon Boehringer Ingelheim Co., Ltd., Tokyo, Japan; 6 PMS Division, Statistics Analysis Department 2, EPS Corporation, Tokyo, Japan; 7 Post Marketing Surveillance Group, Pharmacovigilance, Nippon Boehringer Ingelheim Co., Ltd., Tokyo, Japan; 8 Research Institute for Diseases of the Chest, Graduate School of Medical Sciences, Kyusyu University, Fukuoka, Japan

## Correction to: International Journal of Clinical Oncology 10.1007/s10147-019-01439-5

The article Real-world treatment of over 1600 Japanese patients with EGFR mutation-positive non-small cell lung cancer with daily afatinib, written by Kazuo Tamura, Toshihiro Nukiwa, Akihiko Gemma, Nobuyuki Yamamoto, Masaya Mizushima, Kaori Ochai, Rie Ikeda, Hisaya Azuma, and Yoichi Nakanishi was originally published electronically on the publisher’s internet portal (currently SpringerLink) on 5 April 2019 without open access.

 With the author(s)’ decision to opt for Open Choice the copyright of the article changed on 15 June 2019 to © The Author(s) 2019 and the article is forthwith distributed under the terms of the Creative Commons Attribution 4.0 International License (http://creativecommons.org/licenses/by/4.0/), which permits use, duplication, adaptation, distribution and reproduction in any medium or format, as long as you give appropriate credit to the original author(s) and the source, provide a link to the Creative Commons license and indicate if changes were made.

