# Supplementary material for: Real-world treatment of over 1600 Japanese patients with EGFR mutation-positive non-small cell lung cancer with daily afatinib
Source: Int J Clin Oncol. 2019 Apr 5;24(8):917–26. doi: 10.1007/s10147-019-01439-5 (PMC6597604; doi:10.1007/s10147-019-01439-5)
Supplement: Supplementary file 1 — Supplementary material 1 (DOCX 12 kb) [file 10147_2019_1439_MOESM1_ESM.docx]

**Supplementary Table** ADRs^a^ of special interest by age subgroup

| Safety set | |  | |  |  | |
| --- | --- | --- | --- | --- | --- | --- |
|  | Age group, years | | N | All, n (%) | | Grade ≥3, n (%) |
| **Any ADR** |  | |  |  | |  |
|  | <75 | | 1292 | 1236 (95.7) | | 482 (37.3) |
|  | ≥75 | | 307 | 286 (93.2) | | 98 (31.9) |
| **Diarrhea** |  | |  |  | |  |
|  | <75 | | 1292 | 1046 (81.0) | | 209 (16.2) |
|  | ≥75 | | 307 | 208 (67.8) | | 32 (10.4) |
| **Rash/acne^b^** |  | |  |  | |  |
|  | <75 | | 1292 | 798 (61.8) | | 74 (5.7) |
|  | ≥75 | | 307 | 138 (45.0) | | 19 (6.2) |
| **Nail effects^b^** |  | |  |  | |  |
|  | <75 | | 1292 | 507 (39.2) | | 54 (4.2) |
|  | ≥75 | | 307 | 93 (30.3) | | 11 (3.6) |
| **ILD^b^** |  | |  |  | |  |
|  | <75 | | 1292 | 52 (4.0) | | 32 (2.5) |
|  | ≥75 | | 307 | 18 (5.9) | | 8 (2.6) |

ADR, adverse drug reaction

^a^According to Common Terminology Criteria for Adverse Events (NCI-CTCAE) version 3.0, ^b^Grouped term.
